# Analysis of Alternative Splicing and Alternative Polyadenylation in *Populus alba* var. *pyramidalis* by Single-Molecular Long-Read Sequencing

**DOI:** 10.3389/fgene.2020.00048

**Published:** 2020-02-07

**Authors:** Hongyin Hu, Wenlu Yang, Zeyu Zheng, Zhimin Niu, Yongzhi Yang, Dongshi Wan, Jianquan Liu, Tao Ma

**Affiliations:** ^1^State Key Laboratory of Grassland Agro-Ecosystem, Institute of Innovation Ecology & School of Life Sciences, Lanzhou University, Lanzhou, China; ^2^Key Laboratory of Bio-Resource and Eco-Environment of Ministry of Education, College of Life Sciences, Sichuan University, Chengdu, China

**Keywords:** *Populus alba* var. *pyramidalis*, single molecule real-time technology sequencing, alternative splicing, alternative polyadenylation, long non-coding ribonucleic acid, fusion genes

## Abstract

Poplars are worldwidely cultivated with ecologically and economically important value. *Populus alba* var. *pyramidalis* (= *P*. *bolleana*) is a main tree of the farmland shelter-belt system in the arid region of Northwest China due to its rapid growth, erect stems, and high biomass production. However, the full-length messenger RNA (mRNA) sequences and complete structure of *P. alba* var. *pyramidalis* remain unclear. In this study, using single-molecular real-time (SMRT) and next-generation high-throughput sequencing (NGS) platform, we sequenced transcripts from leaf, root, xylem, and phloem of *P. alba* var. *pyramidalis*, to obtain the full-length mRNA transcripts and annotate the complete structure. In total, 86,327 mapped full-length non-chimeric (FLNC) reads were identified, with 705 previously unannotated loci and 3,410 long noncoding RNAs (lncRNAs) and 174 fusion genes found. Alternative spicing (AS) events were detected in 7,536 genes, of which 4,652 genes had multiple AS events. A total of 10,213 alternative polyadenylation (APA) sites were identified, with two or more APA sites observed in 2,212 genes. Our transcriptome data provided the full-length sequences and gene isoforms of transcripts for *P. alba* var. *pyramidalis*, which will be helpful in improving our understanding for the genome annotation and gene structures of *P. alba* var. *pyramidalis*.

## Introduction

Poplars (*Populus* spp.) are widely used as a model system for tree biology research due to their rapid growth, ease of cloning and genetic transformation, moderate genome size, and extensive genetic diversity (Bradshaw et al., 2000; Jansson and Douglas, 2007). Over the past two decades, a large number of genomic and molecular biology resources of this model plant have been developed. For example, the genomes of various poplar species, including *Populus trichocarpa*, *Populus euphratica*, *Populus tremula* and so on, have been sequenced (Tuskan et al., 2006; Ma et al., 2013; Yang et al., 2017; Lin et al., 2018; Liu et al., 2019; Ma et al., 2019). At the same time, poplars have also been extensively studied at the transcriptome (Qiu et al., 2011; Zhang et al., 2014), proteomics (Xiao et al., 2009), methylome (Song et al., 2015; Su et al., 2018), and population genomics levels (Evans et al., 2014; Ma et al., 2018; Wang et al., 2020), focusing on long-term perennial growth, wood development, flowering, sex determination, and adaptation to environmental stress. Obviously, these resources have enabled rapid progress in our understanding of forest tree growth, development, and evolution of adaptive traits.

However, it is worth noting that almost all of these studies rely on the accurate prediction of transcript sequences and structure. As a powerful tool for gene model annotation and quantification of gene expression levels, high-throughput RNA-sequencing is therefore increasingly important for these studies. Recent researches have shown that the post-transcriptional regulation including alternative splicing (AS) and alternative polyadenylation (APA) contribute significantly to enhance transcriptome diversity in eukaryotic organisms (Reddy, 2007; Kalsotra and Cooper, 2011; Elkon et al., 2013). This transcriptome complexity plays an important role in regulating gene expression during development or in responses to environmental stress (Barbazuk et al., 2008; Chamala et al., 2015; Sun et al., 2016). However, accurate detection of AS and APA events remains a challenge due to the limitations of short-read sequences in reconstructing full-length isoforms (Alkan et al., 2011). These disadvantages generally lead to gene prediction without reliable annotation on alternative isoforms and untranslated regions, which would limit their use to characterize the post-transcriptional processes (Chen et al., 2017). In addition, short-read sequencing sometimes produces low-quality transcripts, resulting in incorrect annotations (Wang et al., 2016). Therefore, the identification of full-length splice isoforms, as well as accurate and complete annotation of genome are essential for a deep understanding of the transcriptome complexity and its potential role in gene regulation.

Single-molecule real-time (SMRT) sequencing, also called the third-generation sequencing technology developed by Pacific Biosciences (PacBio), allows direct sequencing of full-length complementary DNA (cDNA) sequences and avoids the transcriptome assembly that is required for short-read sequencing (Sharon et al., 2013; Peng et al., 2016; Tombacz et al., 2017). Use of SMRT sequencing permits efficient analysis of exon-intron structure and accurate identification of full-length splice isoforms and APA sites, thus facilitating a complete understanding of the transcriptome diversity. Recently, single molecule sequencing technology has been successively used to characterize the complexity of transcriptome in *Sorghum bicolor* (Abdel-Ghany et al., 2016), *Zea mays* (Wang et al., 2016), *Fragaria vesca* (Li et al., 2017), *Phyllostachys edulis* (Wang et al., 2016).

*Populus alba* (white poplar) is one of the most important ecological and economic poplar species, which is widely distributed and cultivated in Central Asia and Europe (Stettler et al., 1996; Ma et al., 2019). Previously we reported the genome sequence of one of its variety, var. *pyramidalis* (= *P. bolleana*) (Ma et al., 2019), which has been widely used for ecological restoration and urban afforestation in northern China by cloning and breeding of branch cuttings (Zhang et al., 2008; Xu et al., 2011). In addition, we also confirmed its high transformation efficiency and short transformation time by experiments. These findings make this species a new candidate model for genetic transformation and gene function studies in poplar tree species (Ma et al., 2019). However, it is still difficult to obtain full-length cDNA for gene annotation, and the transcriptome diversity caused by AS and APA remains unclear in this species. Here, we performed SMRT sequencing to generate a full-length transcriptome in *P. alba* var. *pyramidalis*. To ensure extensive coverage of transcript isoforms, we multiplexed four tissues (leaf, phloem, xylem, and root) and pooled them for transcriptome sequencing by SMRT. In parallel, messenger RNA from these tissues were also sequenced on the Illumina HiSeq 2500 platform to evaluate the isoform expression levels. Based on the obtained full-length transcripts, we improved genome annotation and identified multiple AS and APA events along with the expression patterns of the AS events in various tissues. Our results provide comprehensive information on post-transcriptional regulation that will facilitate future research in poplar.

## Materials and Methods

### Plant Materials and RNA Preparation

Two-year-old *P. alba* var. *pyramidalis* seedlings were collected from Akesu, Xinjiang province, China and planted in pots with loam soil. All seedlings were grown in a greenhouse with a photoperiod of 16 h light/8 h darkness (6:30–22:30) and 60% humidity. For RNA sequencing, total RNA was extracted from leaf, phloem, xylem, and root tissues from each seedling using the cetyl trimethylammonium bromide (CTAB) procedure (Chang et al., 1993). Each sample was performed in triplicate using three individual seedlings treated under the same conditions. Samples were collected and the integrity and quality of the RNA samples were examined with a NanoDrop 8000 UV-Vis Spectrophotometer (Thermo, Darmstadt, Germany). The A260/A280 ratio of the RNA samples was between 1.9 and 2.1, and the RNA integrity number (RIN) values ranged from 8.6 to 10.0.

### PacBio Library Construction and Sequencing

Total RNA from the four different tissues were mixed equally for the PacBio library construction. The library was prepared according to Isoform Sequencing (Iso-Seq) protocol, as described by Pacific Biosciences. Briefly, the first-strand cDNA was synthesized using a Clontech SMARTer PCR cDNA Synthesis Kit. After the PCR amplification, the products were purified with AMPurePB magnetic beads. The concentration and size of the purified products were detected using the Qubit 2.0 Fluorometer (Life Technologies) and Agilent 2100 Bioanalyzer (Agilent Technologies). Then, the 1–2 kb, 2–3 kb, and > 3 kb cDNA fractions were generated with a BluePippin size selection system (Sage Science, http://www.sagescience.com/). The three libraries were constructed with a Pacific Biosciences SMRTbell Template Prep Kit 1.0 according to the manufacturer's instruction. The libraries were subsequently sequenced on the PacBio RS II real-time (RT) sequencer platform with a total of six SMRT cells: the 1–2 kb library was sequenced using two SMRT cells, while the 2–3 kb library used three SMRT cells and the > 3 kb library used one SMRT cell. In total, 10.46 Gb subreads were produced with depth of ~22.5× based on genome size of 464 M (**Table S1**). The PacBio SMRT sequencing data have been submitted to the Sequence Read Archive (SRA) of NCBI under accession number SRR5990031.

### Illumina RNA-Sequencing Library Construction and Sequencing

The Illumina HiSeq 2500 platform was used to generate paired-end (PE) reads to correct PacBio reads and quantify splicing. Strand-specific RNA-seq libraries were constructed using NEB Next Ultra Directional RNA Library Prep Kit for Illumina according to the manufacturer's instructions. Libraries were controlled for quality and quantified using the Bioanalyzer 2100 system and quantitative PCR (qPCR). The resulting libraries were finally sequenced on a HiSeq 2500 sequencing system as 125-nt paired-end reads. The Illumina HiSeq 2500 data have been submitted to the Sequence Read Archive (SRA) of NCBI under accession number SRX3504248-SRX3504283.

### Analysis of PacBio Single-Molecule Long-Reads

*P. alba* var. *pyramidalis* genome sequence and annotated gene models were downloaded from Genome Warehouse in BIG Data Center (accession number GWHAAEP00000000) (Ma et al., 2019). ConsensusTools from the smrtanalysis_2.3.0 (Pacific Biosciences) was used from the command line to get reads of the insert (ROI). Then the full-length non-chimeric (FLNC) transcripts were determined by searching for the polyA tail signal and the 5′ and 3′ cDNA primers in the ROIs. Then high quality full-length (FL) transcripts were further corrected using the Illumina RNA-seq data with the software LoRDEC under default setting -k 23, -s 3 (Salmela and Rivals, 2014). Finally, the obtained high-quality FLNC reads were then mapped to the reference genome of *P. alba* var. *pyramidalis* using Genomic Mapping and Alignment Program (GMAP) with the following option: –cross-species –no-chimeras –min-trimmed-coverage = 0.85 –min-identity = 0.9 (Wu and Watanabe, 2005). The BUSCO was used to evaluate the integrity of the transcriptome without redundancy, and the number of embryophyta gene sets used in this evaluation was 1,440 (Simao et al., 2015).

Novel genes and alternative polyadenylation (APA) sites were identified by TAPIS (Abdel-Ghany et al., 2016). We used the reads that aligned to annotated genes for the analysis of poly(A) sites, the depth of which was calculated as the number of reads aligning within a window of five nucleotides (nt) of the candidate poly(A) site, and a window of 15 nt was used to cluster micro-heterogeneity sites. Multiple expectation maximization for motif elicitation (MEME) was subsequently used for motif searches on the upstream and downstream sequences of poly(A) sites (Timothy, et al., 2009). The reads that overlapped no annotated genes were classified as novel genes.

### Identification of Fusion Transcripts and Alternative Splicing From PacBio Sequences

A python script (fusion_finder.py) in the cDNA_Cupcake package (https://github.com/Magdoll/cDNA_Cupcake) was used to identify fusion transcripts. Each candidate fusion transcript must be mapped to two or more loci in the reference genome with an interval of at least 10 kb, and each locus must cover at least 10% of the transcript. The total coverage of the fusion transcripts was at least 99%. To further exclude putative false candidates, transcripts involving two or more genes from the same gene family were discarded.

The alternative splicing (AS) events, including intron retention (IR), exon skipping (ES), alternative 5' splice site (Alt 5'), and alternative 3' splice site (Alt 3') were identified by the TAPIS pipelines (Abdel-Ghany et al., 2016). The differential AS events were detected using the program rMATS (Shen et al., 2014) based on the Illumina RNA-seq data.

### Identification of Long Non-Coding RNAs From PacBio Sequences

Four computational approaches, including coding potential calculator (CPC), coding-non-coding index (CNCI), the predictor of long non-coding RNAs, and messenger RNAs based on an improved k-mer scheme (PLEK), and the Pfam database were combined to sort non-protein coding RNA candidates from putative protein-coding RNAs in the transcripts (Wang et al., 2018). The putative protein-coding RNAs were filtered out and the transcripts with lengths greater than 300 nt were selected as lncRNA candidates (Frith et al., 2006; Dinger et al., 2008; Niazi and Valadkhan, 2012). The putative lncRNAs were further screened using CPC, CNCI, PLEK, and Pfam, and the common results obtained by these four approaches were used for the subsequent analysis.

### Illumina RNA-Sequencing Data Analysis

First, clean reads were obtained by removing reads containing the adapter, reads containing unknown bases (> 10%), and low-quality reads (when the percentage of low-quality bases was over 50% in a read) from raw reads. At the same time, the Q20, Q30, guanine-cytosine (GC)-content, and sequence duplication level of the clean reads were calculated. The clean reads were then mapped to the reference genome using Hisat2 (Kim et al., 2015). Only those reads with a perfect match or one mismatch were further analyzed and annotated based on the reference genome. Fragments Per Kilobase of transcripts per Million fragment mapped (FPKM) was used in calculating the expression level of genes or transcripts. Differential expression analysis between the two tissues was performed using the DESeq2 R package (Love et al., 2014). Genes with an adjusted P-value < 0.05, and fold change (FC) ≥2 (|log2 (fold change)| ≥ 1) identified by DESeq2 were assigned as differentially expressed.

### Gene Ontology Enrichment Analysis

The GO terms of *P. alba* var. *pyramidalis* were assigned to each gene based on InterProScan (Jones et al., 2014), and then used the R packages of ClusterProfile (Yu et al., 2012) to perform GO enrichment analysis.

### Quantitative Real-Time Polymerase Chain Reaction

To verify the reliability of the RNA-Seq analyses, nine genes were selected for quantitative real-time PCR (qRT-PCR) analysis. A portion (0.5 µg) of DNase I-treated total RNA from 12 samples was transformed into single-stranded cDNA with a PrimeScript 1st Strand cDNA Synthesis kit (Takara, Dalian, China). The cDNA templates were diluted 20-fold and amplified with a CFX96 Real-Time PCR Detection System (Bio-Rad, Singapore) and SYBR Premix ExTaq™ (Takara). The templates were amplified using the following program: 95°C for 15 s, 60°C for 30 s, and finally 72°C for 20 s. The primers were designed using the Primer Premier 5 software (PREMIER Biosoft, Palo Alto, CA, USA) and are listed in **Data S1**. Three biological replicates were used for each gene. The relative expression levels of the genes, which were normalized to the expression of the internal reference gene actin, were calculated according to the 2*−*ΔΔct method (Livak and Schmittgen, 2001).

## Results and Discussion

### Single Molecule Real-Time Technology Sequencing and Read Mapping

We collected RNA samples from four tissues, including leaf, phloem, xylem, and root and then equally pooled them together to acquire full-length transcripts for single-molecule long-read sequencing (pipeline in **Figure S1**). Three different libraries, with cDNA insert size 1–2, 2–3, and > 3 kb respectively, were constructed and sequenced using a PacBio RSII sequencing platform. Six single molecular real-time (SMRT) cells generated a total of 121,487, 138,596, and 60,387 reads of inserts (ROIs) from these three libraries respectively, the length distribution of which were consistent with their expected insert size (**Table 1**; **Figure S2**). After removing adaptor sequences, low-quality sequences, and short sequences (< 50 bp), a total of 319,689 sub-reads were remained, and more than 74% (235,627) of them were identified as FLNC reads with the entire transcript region from the 5' to the 3' end based on the inclusion of barcoded primers and the 3' poly(A) tails. The average length of these FLNC reads was 2,341 bp (**Table 1**). In parallel, three biological replicates for each sample were used for the Illumina RNA-seq library construction and sequencing. These short reads were subsequently used to further correct the FLNC by LoRDEC (Salmela and Rivals, 2014), quantify gene expression and detect AS events. Finally, a total of 104,755 unique corrected SMRT transcripts were obtained, of which 58.44% were longer than 2 kb (**Table S2**).

**Table 1:** Summary of PacBio single-molecule long-read sequencing.

	1–2 kb	2–3 kb	3–6 kb
No. of reads of consensus reads	121,487	138,596	60,387
No. of 5` reads	101,789	112,789	48,970
No. of 3` reads	104,914	115,391	50,463
No. of poly(A) reads	104,556	113,195	50,346
No. of filtered short reads	124	143	17
No. of non-full-length reads	27,438	39,969	16,328
No. of full-length reads	93,925	98,157	44,042
No. of full-length non-chimeric reads	93,619	98,089	43,919
Average full-length non-chimeric read length	11,89	2,335	3,498

To further test the completeness of our transcriptome, we used the Benchmarking Universal Single-Copy Orthologs (BUSCO) pipeline to compare our *P. alba* var. *pyramidalis* transcriptome to 1,440 conserved embryophyta genes. The results showed that 1,052 (73.1%) genes could be completely covered by our transcriptome data. Of these, 35.7% (514 genes) and 37.4% (538 genes) were complete single-copy and duplicated BUSCOs respectively, while 5.9% (85 genes) and 21.0% (303 genes) were fragmented and missed BUSCOs, respectively.

**Figure 1:**
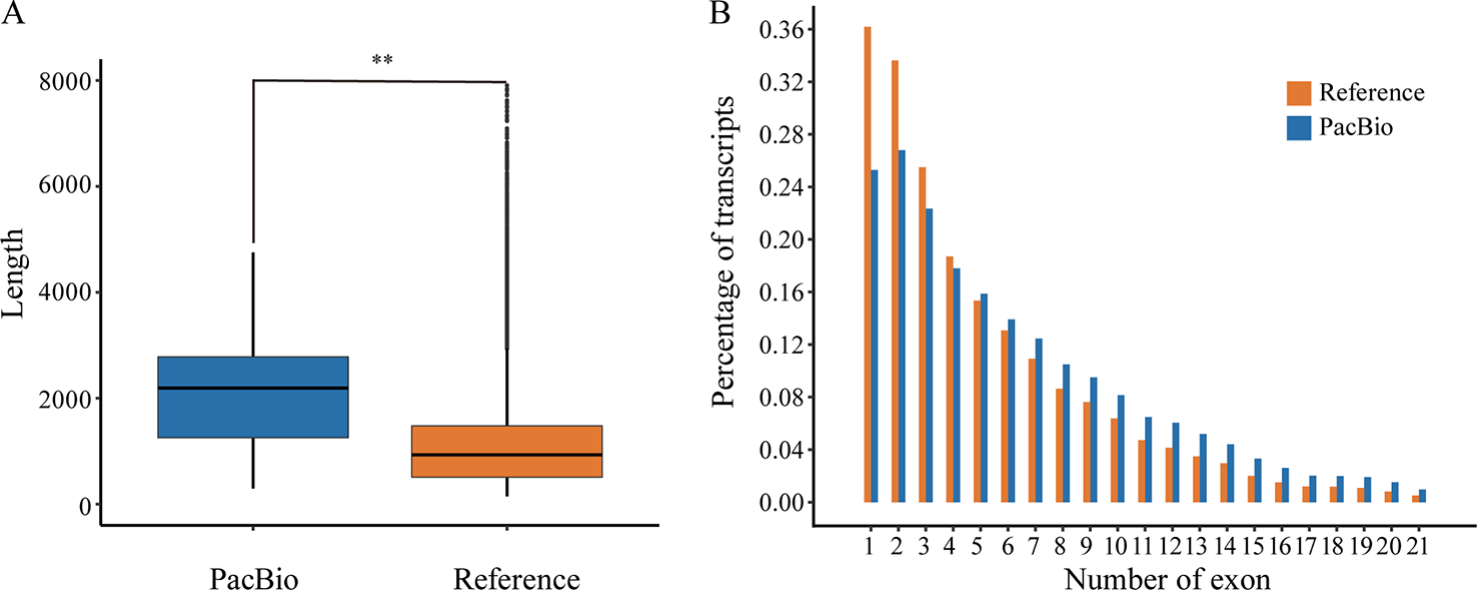
The comparison between reference annotation and PacBio Iso-Seq data. **(A)** Box-plots show distribution patterns of transcript length (Wilcoxon rank sum test:**, P < 0.001). **(B)** Distribution of exon number per isoform.

The corrected FLNC reads were then mapped against the reference genome of *P. alba* var. *pyramidalis* by GMAP (Wu and Watanabe, 2005). The results showed that 82.41% (86,327) of the reads could be reliably determined. After being compared with previous annotation, these mapped reads covered a total of 15,173 (40.03%) annotated genes, among which 6,881 genes were supported by at least two FLNC reads. We also found that the full-length transcripts (mean 2,136 bp) were generally longer (**Figure 1A**) than reference transcripts (mean 1,146 bp) and covered a large number of genes with multiple exons (**Figure 1B**).

### Identification of Novel Genes, Long Non-Coding RNA, and Fusion Transcripts

We further assessed the completeness of current gene annotation. Those which could not be aligned were considered as novel transcripts (Wang et al., 2017). In total, 2,464 FLNC reads have no overlap with any annotated gene, which are likely transcripts come from novel loci (**Data S2**). These FLNC reads were combined into 705 consensus clusters, among which 2,372 had known homologous in the BLAST search against SwissProt, UniPort90, or InterPro protein databases (**Figure S3**). These clusters likely represent transcripts from novel protein-coding genes in *P. alba* var. *pyramidalis*. On the whole, these novel transcripts displayed lower expression and shorter length than other transcripts, which might be the reasons that why these transcripts were excluded from previous annotation (**Figure S4**).

We also used four tools, including CPC, CNCI, Pfam, and PLEK analysis (Wang et al., 2018), to identify unique transcripts without protein coding potential (**Figure S5A**). In total, 3,410 unique transcripts were identified to be putative long non-coding RNAs (lncRNAs), of which 1,622 (47.6%) were single exon (**Figure S5B**). These lncRNAs had a mean length of 1,267 bp ranging from 300 to 4,346 bp. We classified them into four groups based on their positions relative to protein-coding gene annotations: 719 of them were generated from intergenic regions (lincRNA), 385 from the antisense strand (antisense-lncRNA), 2,211 from the sense strand (sense-lncRNA), and 58 from intronic regions (Intronic-lncRNA) (**Figure S5C**). We furtherly detected 59 AS and 65 APA events for all of the lncRNA. Only 6% lncRNA genes exists AS events and most lncRNA genes just have one polyadenylation site. Compared with protein-coding transcripts, there is no distinct tendency between them in the AS and APA choices (**Figure S6**). However, the proportion of AS and APA genes in long non-coding genes much lower than coding genes (**Table S3**). Expression profiling indicated that these lncRNAs exhibited a tissue-specific expression pattern (**Figure S7A**) and that multi-exon lncRNAs had a higher expression levels than single-exon lncRNAs did (**Figure S7B**).

In addition, we further identified 174 fusion genes using genic alignments, which were formed by merging of two transcripts with different functions (**Data S3**). The majority of fusion transcripts were from different scaffolds (**Figure S8A**). Gene ontology analysis of these fusion transcripts revealed that most were associated with “integral component of Golgi membrane,” “intra-Golgi vesicle-mediated transport,” “structural constituent of cytoskeleton,” and “proton-transporting ATP synthase activity rotational mechanism” (**Figure S8B**). The specific function of the fusion transcripts and lncRNAs identified here will require further investigation in the future.

### Profiling of Global Alternative Polyadenylation Sites

**Figure 2:**
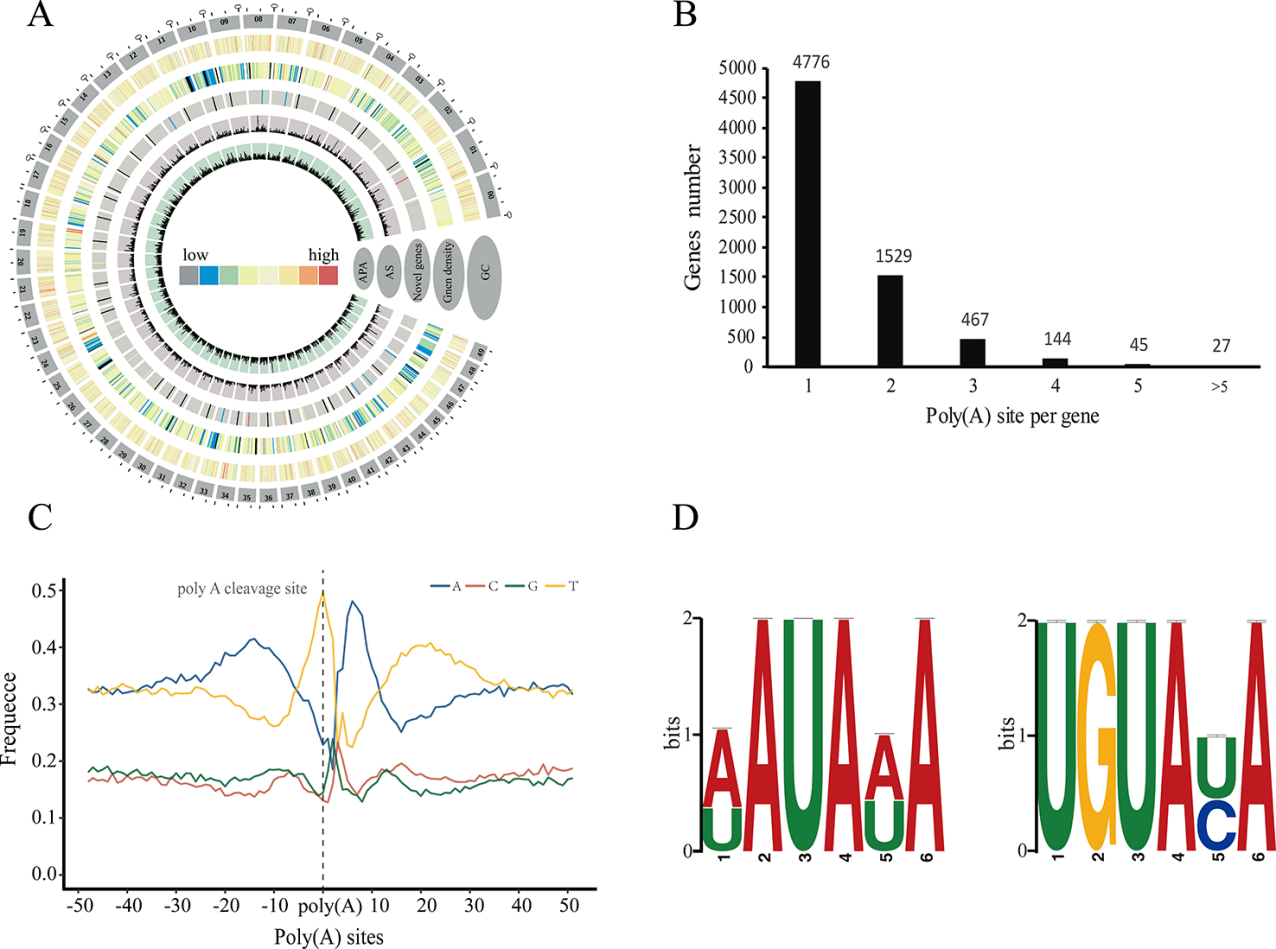
Characteristics of alternative polyadenylation (APA) in *Populus alba* var. *pyramidalis*. **(A)** Concentric circles diagram illustrating the genome-wide profile of novel genes, alternative splicing (AS) and alternative polyadenylation (APA). The largest 50 scaffolds are plotted at the outer circles. From the outer edge to inner circles display guanine-cytosine (GC) content, gene density, novel genes with supported PacBio-isoform number, AS and APA, respectively. **(B)** Distribution of the number of poly **(A)** sites per gene. **(C)** nucleotide composition around poly **(A)** cleavage sites. **(D)** MEME-CHIP analysis identified a poly **(A)** signal in *Populus alba* var. *pyramidalis* transcripts. Left, an over-represented motif about 30 nts upstream of the poly **(A)** site similar to the known signal in dicots was identified. Right, another overrepresented motif (UGUAUA) is found about 50 nts upstream of the poly **(A)** site.

Polyadenylation at the 3' end of messenger RNAs (mRNAs) is one of the most important post-transcriptional modification in eukaryotes, and plays a crucial role in the transport, localization, stability, and translation of transcripts from the nucleus to the cytoplasm (Bentley, 2014). Many studies have shown that transcripts derived from a given gene could contain different poly(A) because of alternative cleavage and polyadenylation, which increases transcriptome complexity and can regulate gene expression through multiple mechanisms in both plants and animals (Shen et al., 2011; Wu et al., 2011; Elkon et al., 2013). Here we used PacBio sequencing to investigate the 3' end of transcripts and identify alternative polyadenylation (APA) events for the first time in *P. alba* var. *pyramidalis* (**Figure 2A**). Of the 15,173 genes detected by our FLNC reads, we identified a total of 10,213 polyadenylation sites from 6,988 genes, of which 2,212 genes were identified with two or more polyadenylation sites (**Figure 2B**). We next analyzed the nucleotide composition in the upstream and downstream 50 bp of all polyadenylation cleavage sites for nucleotide bias. Consistent with findings in other plant species (Abdel-Ghany et al., 2016; Zhu et al., 2017), a clear nucleotide bias was observed in *P. alba* var. *pyramidalis*, with an enrichment of uracil (U) upstream and adenine (A) downstream of the cleavage site (**Figure 2C**). We also performed a MEME analysis to identify potential cis-elements in the upstream 50 nucleotides of the cleavage sites. Two conserved motifs, AAUAAA and UGUA (**Figure 2D**), were finally identified, similar to previous reported patterns in *S. bicolor* and *P. edulis* (Abdel-Ghany et al., 2016; Wang et al., 2017).

**Figure 3:**
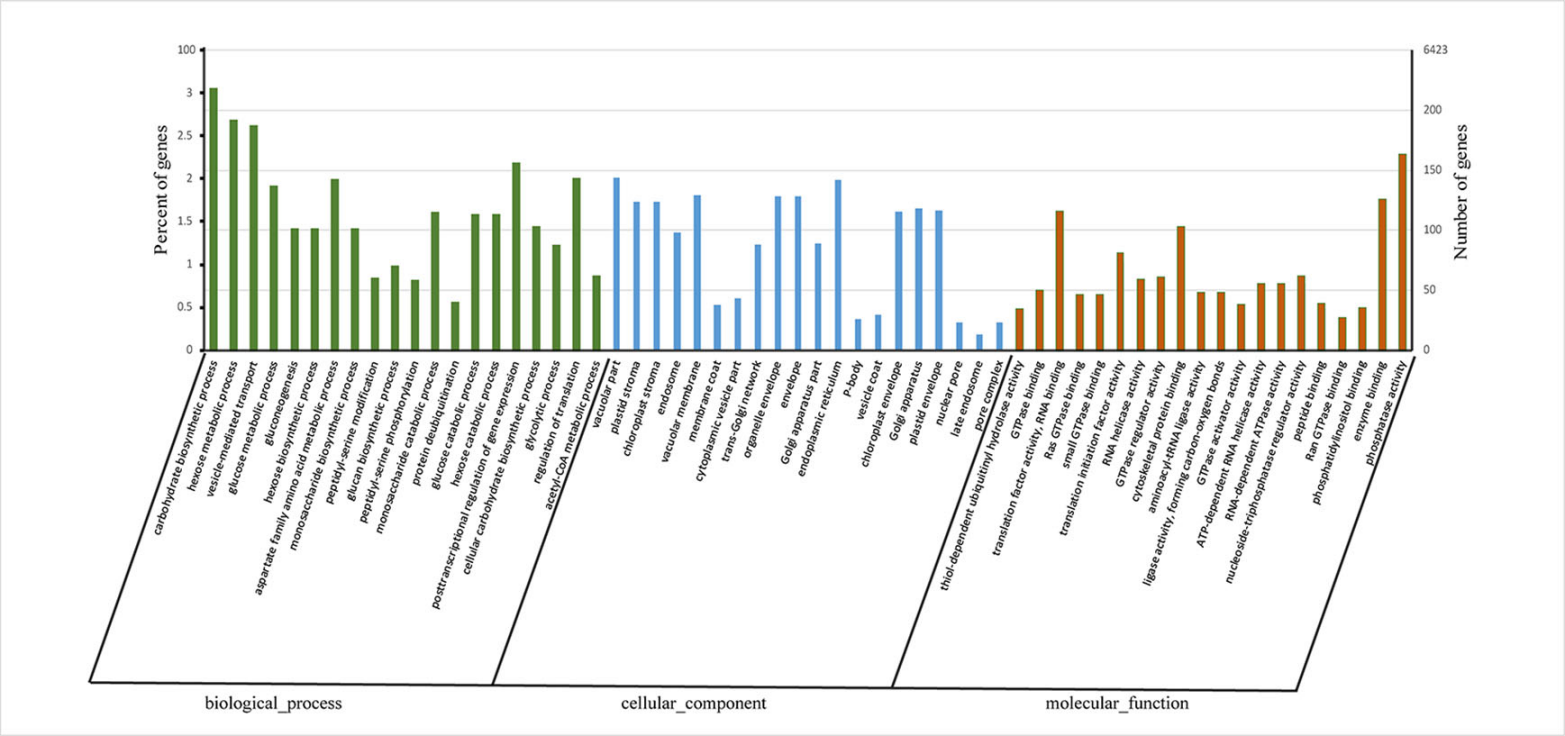
Histogram showing gene ontology (GO) terms in alternative polyadenylation (APA) genes at enrichment significance (qvalue < 0.001 and qvalue top 20 of each category). The X-axis represents GO terms under biological process, cellular component and molecular function. The Y-axis represents enrichment ratio of APA genes.

Gene ontology (GO) enrichment analysis of these APA genes revealed that most were associated with “carbohydrate biosynthetic process,” “hexose metabolic process,” and “vesicle-mediated transport” in biological process category and “thiol-dependent ubiquitinyl hydrolase activity,” “translation factor activity, RNA binding,” and “RNA helicase activity” in molecular function category (**Figure 3**, **Data S4**). These enriched GO terms indicated that APA genes play a major role mainly *via* Influencing energy metabolism and glucose metabolism regulates the synthesis of lignin and cellulose and affects the regulation of transcription factors and the stability of RNA.

### Analysis of Alternative Splicing

**Figure 4:**
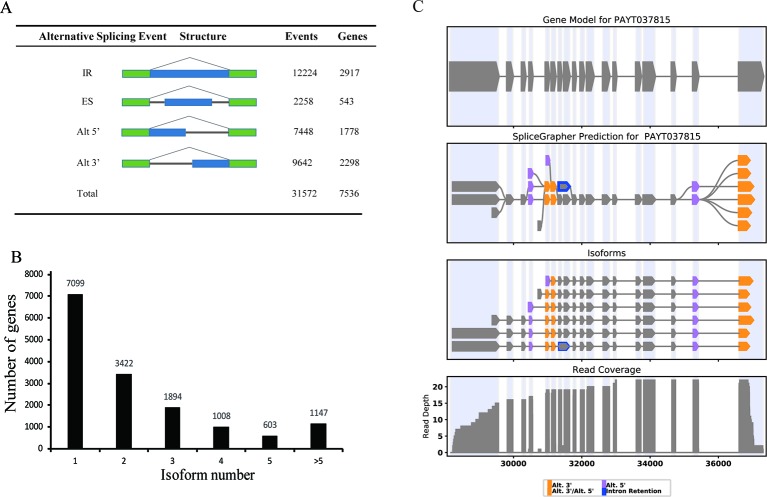
Alternative splicing and splice isoform analysis with Iso-Seq reads. **(A)** The total number of AS events in genes based on Iso-Seq data. Alt 3', alternative 3' splicing; Alt 5', alternative 5' splicing; ES, exon skipping; IR, intron retention; Total, all AS events. **(B)** distribution of genes that produce one or more splice isoform. **(C)** An example of a gene that produces 6 novel splice isoforms. The gene models contain a single splice isoform for this gene. Gene model (top), splice graph (middle), and reads coverage (bottom) are shown.

We compared the PacBio full-length transcripts against the *P. alba* var. *pyramidalis* genome, and detected a total of 31,572 alternative splicing (AS) events. These AS events were further classified into four distinct types: 12,224 (38.7%) intron retention, 2,258 (7.2%) exon skipping events, 7,448 (23.6%) alternative 5'-donor, and 9,642 (30.5%) alternative 3'-donor (**Figure 4A**). Consistent with previous studies in various plants such as sorghum, bamboo, and cotton (Abdel-Ghany et al., 2016; Wang et al., 2017; Wang et al., 2018), intron retention comprised the majority of AS events in *P. alba* var. *pyramidalis*. In our results, only one single isoform was detected in 7,099 genes and two or more isoforms were found in 8,074 genes, which produce a total of 36,378 transcripts. Five or more than five splice isoforms were detected in 1,147 genes (**Figure 4B**). For example, the gene *PAYG037815* was annotated as a single transcriptional gene in current genome annotation, whereas it was detected to produce six isoforms in our study (**Figure 4C**).

**Figure 5:**
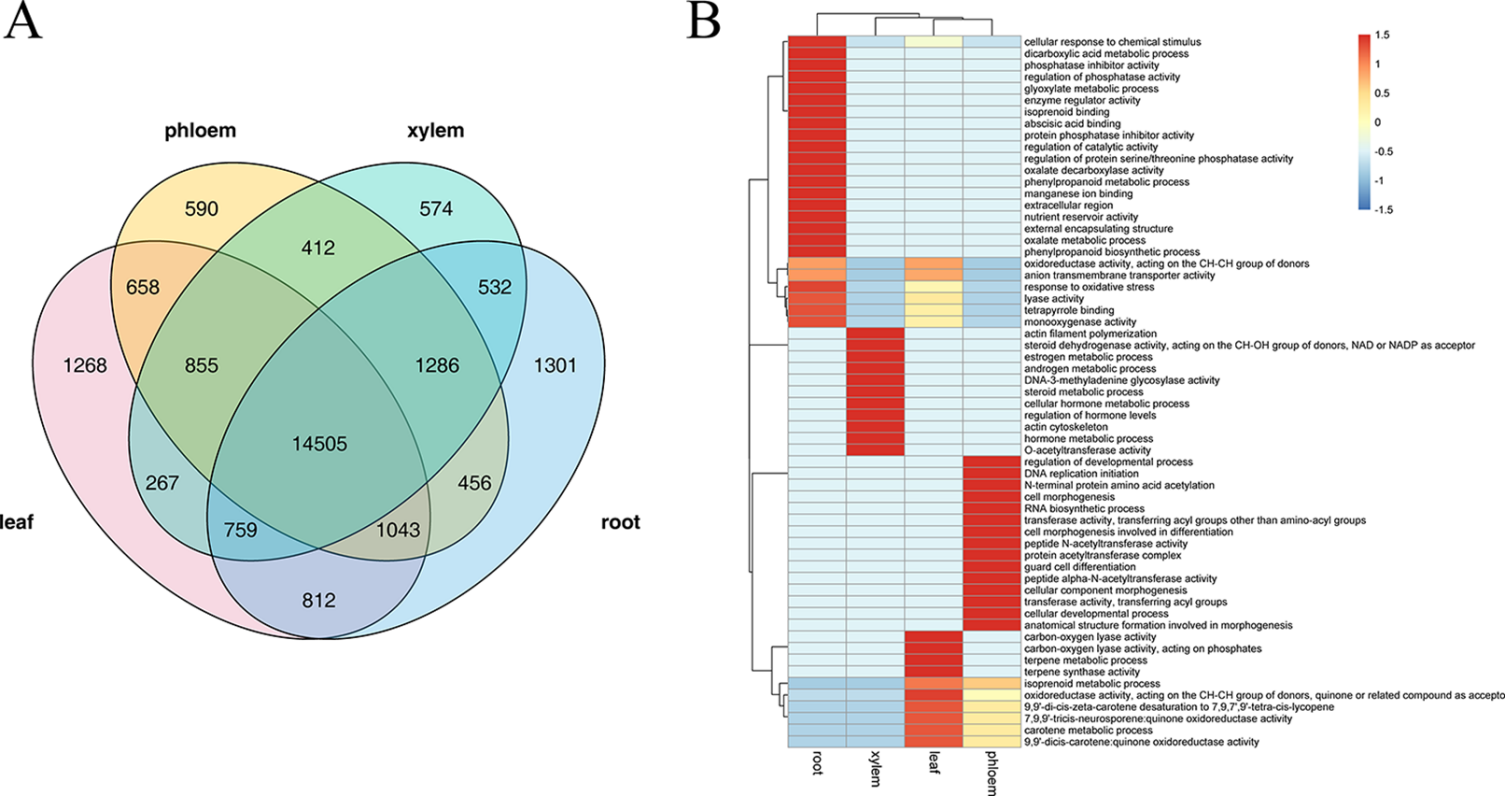
The analysis of tissue-specific alternative splicing (AS) events. **(A)** Venn diagram illustrating the tissue-specific in leaf, phloem, xylem, and root. **(B)** The gene ontology (GO) enrichment heat map for tissue-specific AS genes within the different tissue. GO clustered by row.

Several studies have confirmed that AS is a highly tissue-specific regulation, we therefore identified splicing events specific to leaf, phloem, xylem, and root tissue by combining Illumina sequencing data. Among these tissues, leaf and root tissue had higher proportions of tissue-specific AS events (1,268 and 1,301 respectively), whereas phloem and xylem tissue had lower proportions (590 and 574 respectively) (**Figure 5A**). GO analysis showed that these tissue-specific isoforms are enriched for particular molecular functions that vary with tissue. As shown in **Figure 5B**, “terpene metabolic process,” “7,9,9'-tricis-neurosporene:quinone oxidoreductase activity,” and “carotene metabolic process” were most significant functions of the leaf-specific AS events; “regulation of developmental process,” “cell morphogenesis involved in differentiation,” and “peptide N-acetyltransferase activity” were most significant functions of the phloem-specific AS events; “DNA-3-methyladenine glycosylase activity,” “actin cytoskeleton,” and “regulation of hormone levels” were the most signification functions of the xylem-specific AS events; “cellular response to chemical stimulus,” “abscisic acid binding,” and “regulation of protein serine/threonine phosphatase activity” were the most signification functions of the root-specific AS events. These enriched GO terms indicated that the AS in leaf tissues might play a major role mainly *via* phytochromes activity and photosynthesis, while the AS in root tissue may play a major role mainly through regulate hormone levels and oxidation-reduction processes.

### Validation of Gene Expression Patterns by Quantitative Real-Time Polymerase Chain Reaction

**Figure 6:**
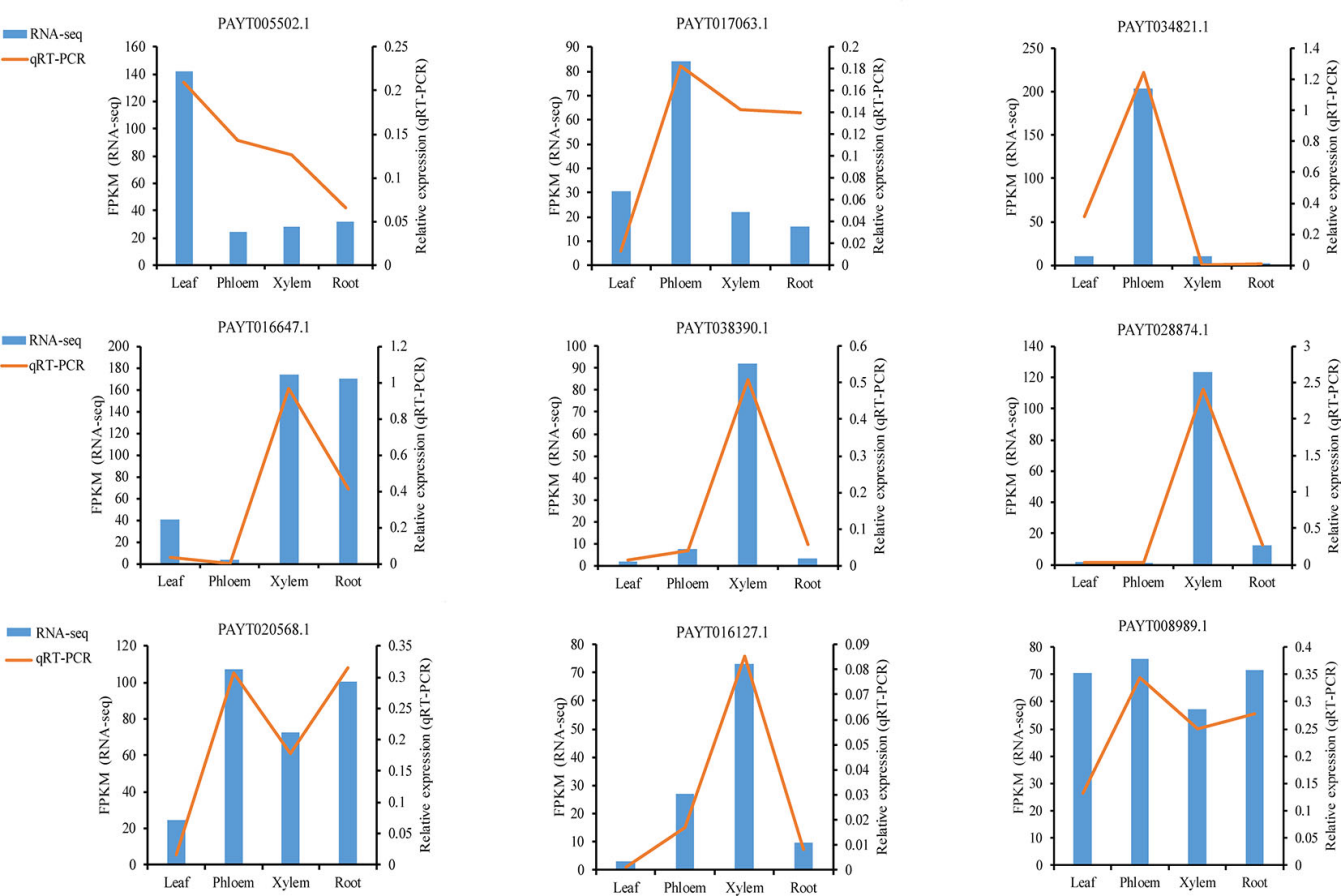
qRT-PCR (quantitative real-time PCR) verification of nine selected genes. Comparison of RNA sequencing (RNA-Seq) data (blue bar) with qRT-PCR data (red line). The normalized expression levels (FPKM) from the RNA-Seq results are indicated on the y-axis to the left. The relative qRT-PCR expression level is shown on the *y*-axis to the right. Actin was used as an internal control. Both methods agree with each other in showing similar gene expression trends.

To validate the gene expression inferred from the RNA-Seq experiments, nine genes were selected for qRT-PCR analysis (**Data S5**). The nine candidate genes included ATP-dependent zinc metalloprotease FTSH 2 (AtFTSH2), protein At1g66480, BURP domain protein RD22, receptor-like protein 44 (AtRLP44), protein BIG GRAIN 1-like B, probable phosphoinositide phosphatase SAC9 (AtSAC9), protein COBRA, glucuronoxylan 4-O-methyltransferase 3, Ras-related protein RABD2a (AtRABD2a). These genes had displayed divergence and significant expression patterns in different tissues. For example, protein BIG GRAIN 1-like B (PAYT038390.1) showed a sharp increase in xylem, indicating that this gene may play a xylem-specific role. Although the FPKM in their expression detected by sequencing did not exactly match those detected by qRT-PCR, the detected expression patterns were mostly consistent for all the selected genes, confirming the reliability of the RNA-Seq results (**Figure 6**).

## Conclusions

In our study, short-read and long-read based mRNA sequencing techniques were applied to obtain the full-length mRNA transcripts and annotate the complete structure of *P. alba* var. *Pyramidalis*. Our resulting transcripts identified 705 new gene loci, 3,410 lncRNAs, and 147 fusion genes. A total of 31,572 AS events were identified. Of which, the intron retention is the major mode of AS in *P. alba* var. *Pyramidalis*. Additionally, a total of 10,213 APA sites were identified and 32% of the mapped genes had multiple sites. Overall, the full-length transcript sequences and complete structure of *P. alba* var. *pyramidalis* will provide an important reference transcript for future researches of *P. alba* var. *pyramidalis*.

## Data Availability Statement

The datasets generated for this study can be found in the Sequence Read Archive (SRA) of NCBI under the accession numbers SRR5990031, SRX3504248 and SRX3504283.

## Author Contributions

TM, JL and DW supervised the project. HH and WY analyzed and interpreted data. HH, WY and YY participated in design and drafting of the manuscript. ZZ and ZN performed the experiments during this study. All authors read and approved the final manuscript.

## Funding

This research was supported by National Key Research and Development Program of China (2016YFD0600101), National Natural Science Foundation of China (31922061, 41871044, 31561123001, 31500502), National Key Project for Basic Research (2012CB114504), and Fundamental Research Funds for the Central Universities (2018CDDY-S02-SCU, SCU2019D013).

## Conflict of Interest

The authors declare that the research was conducted in the absence of any commercial or financial relationships that could be construed as a potential conflict of interest.
