# Supplementary figures and images for: Analysis of Alternative Splicing and Alternative Polyadenylation in Populus alba var. pyramidalis by Single-Molecular Long-Read Sequencing
Source: Front Genet. 2020 Feb 7;11:48. doi: 10.3389/fgene.2020.00048 (PMC7020888; doi:10.3389/fgene.2020.00048)

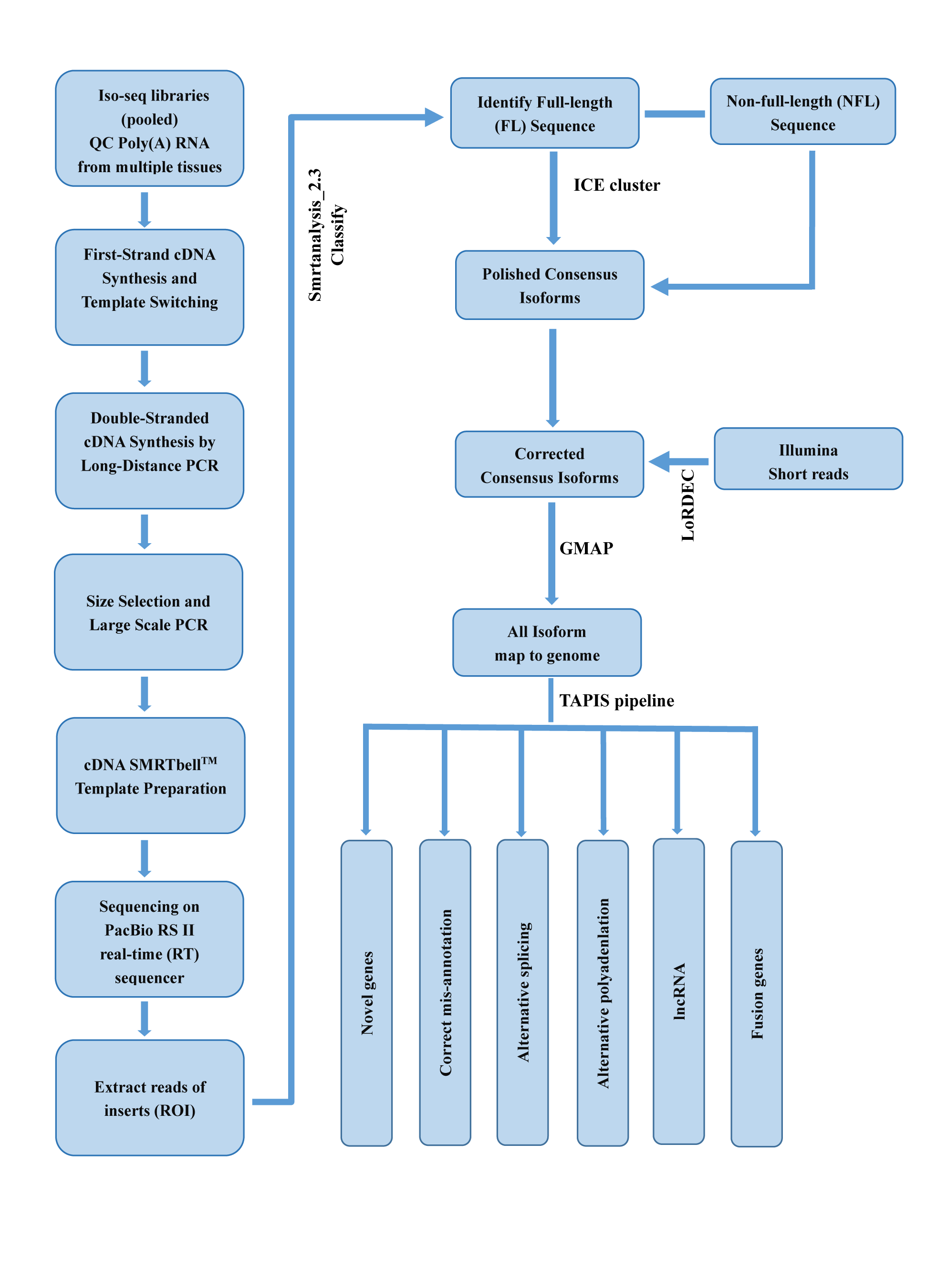

Supplement: Supplementary file 6 [file Image_1.tif]

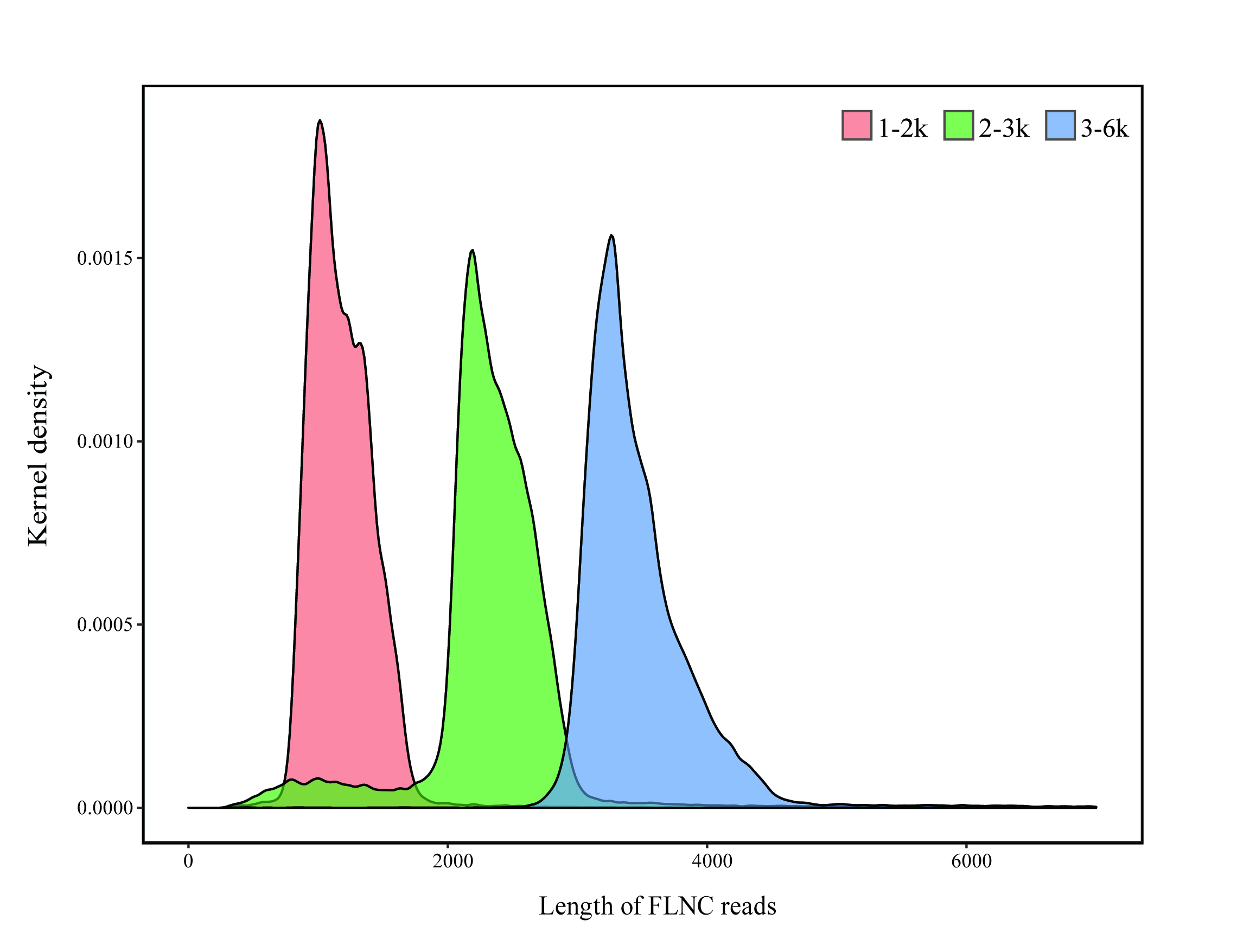

Supplement: Supplementary file 7 [file Image_2.tif]

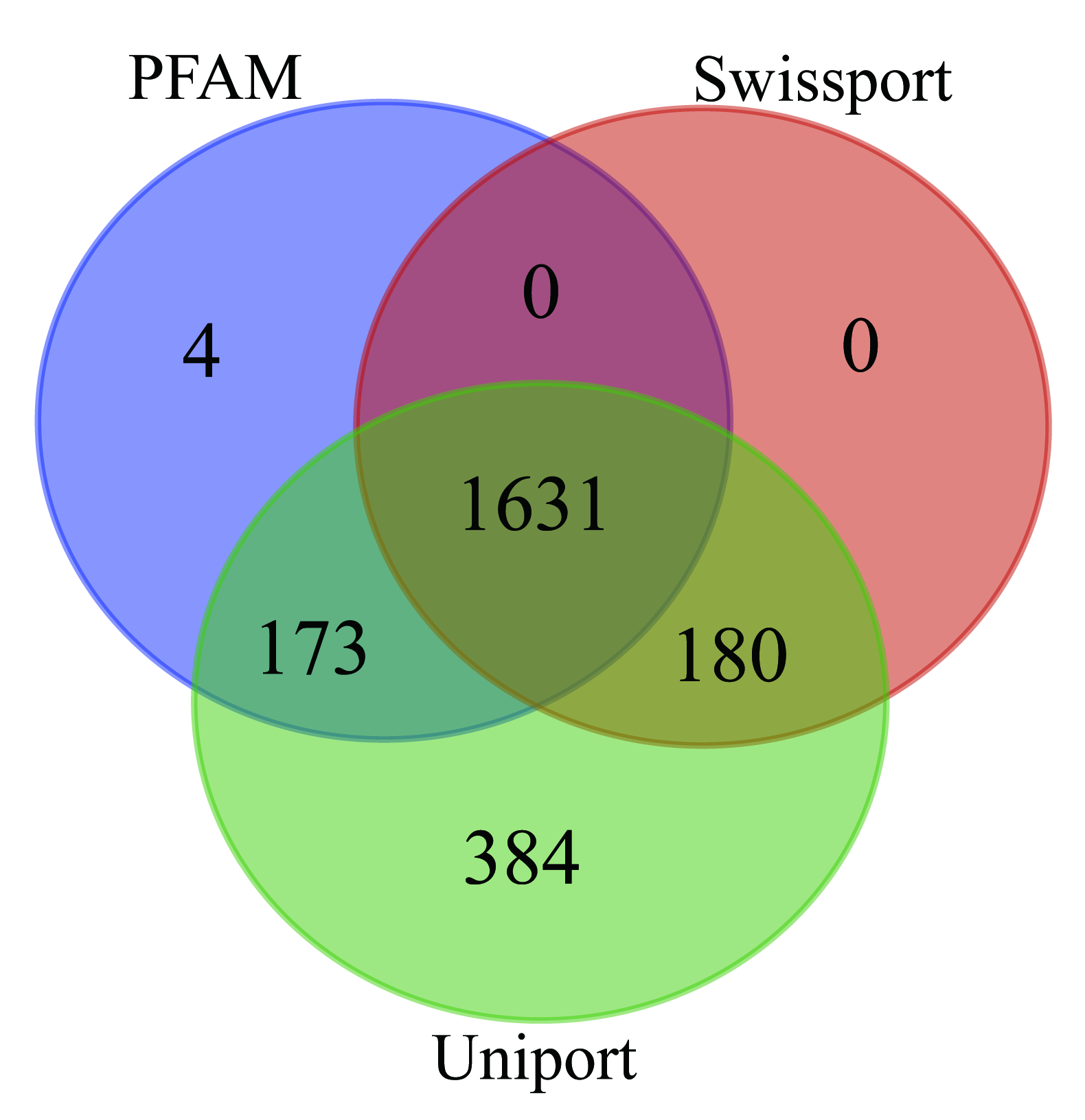

Supplement: Supplementary file 8 [file Image_3.tif]

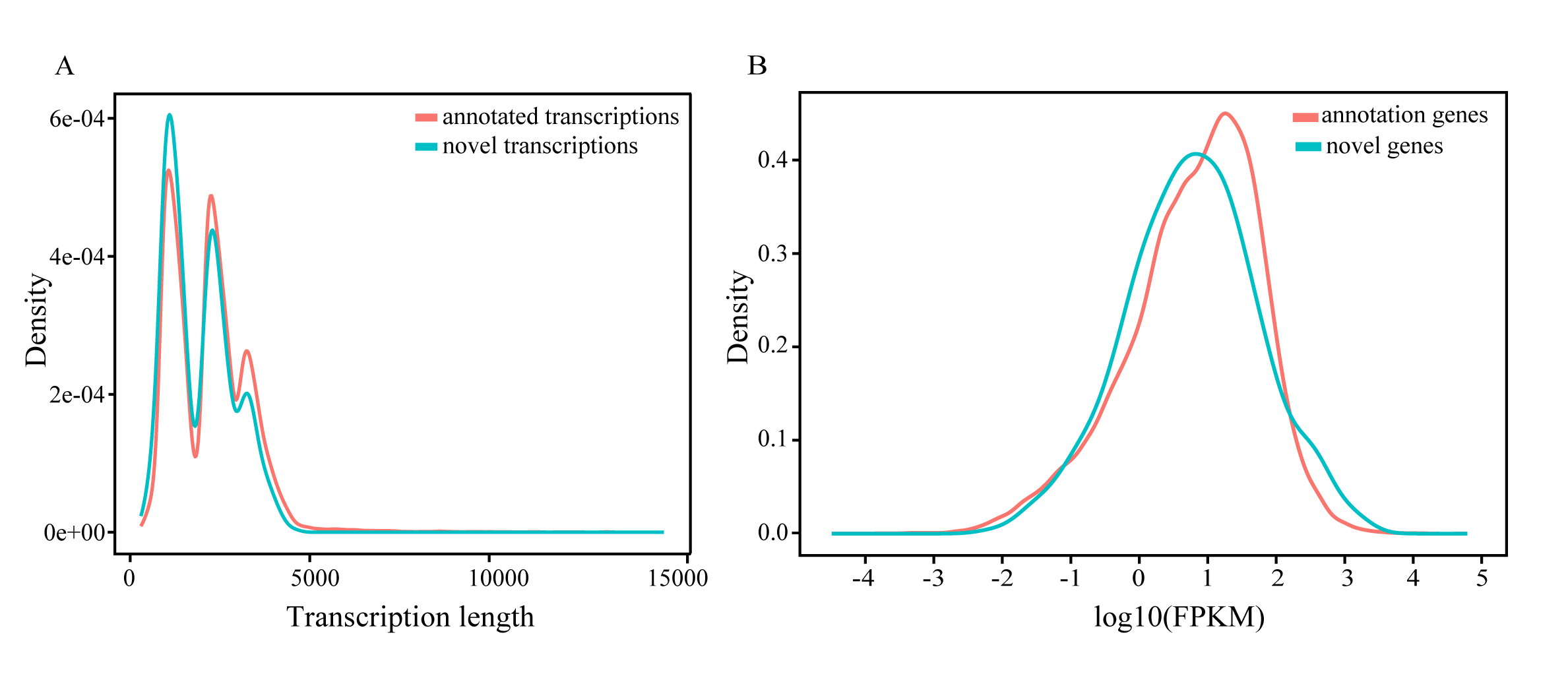

Supplement: Supplementary file 9 [file Image_4.tif]

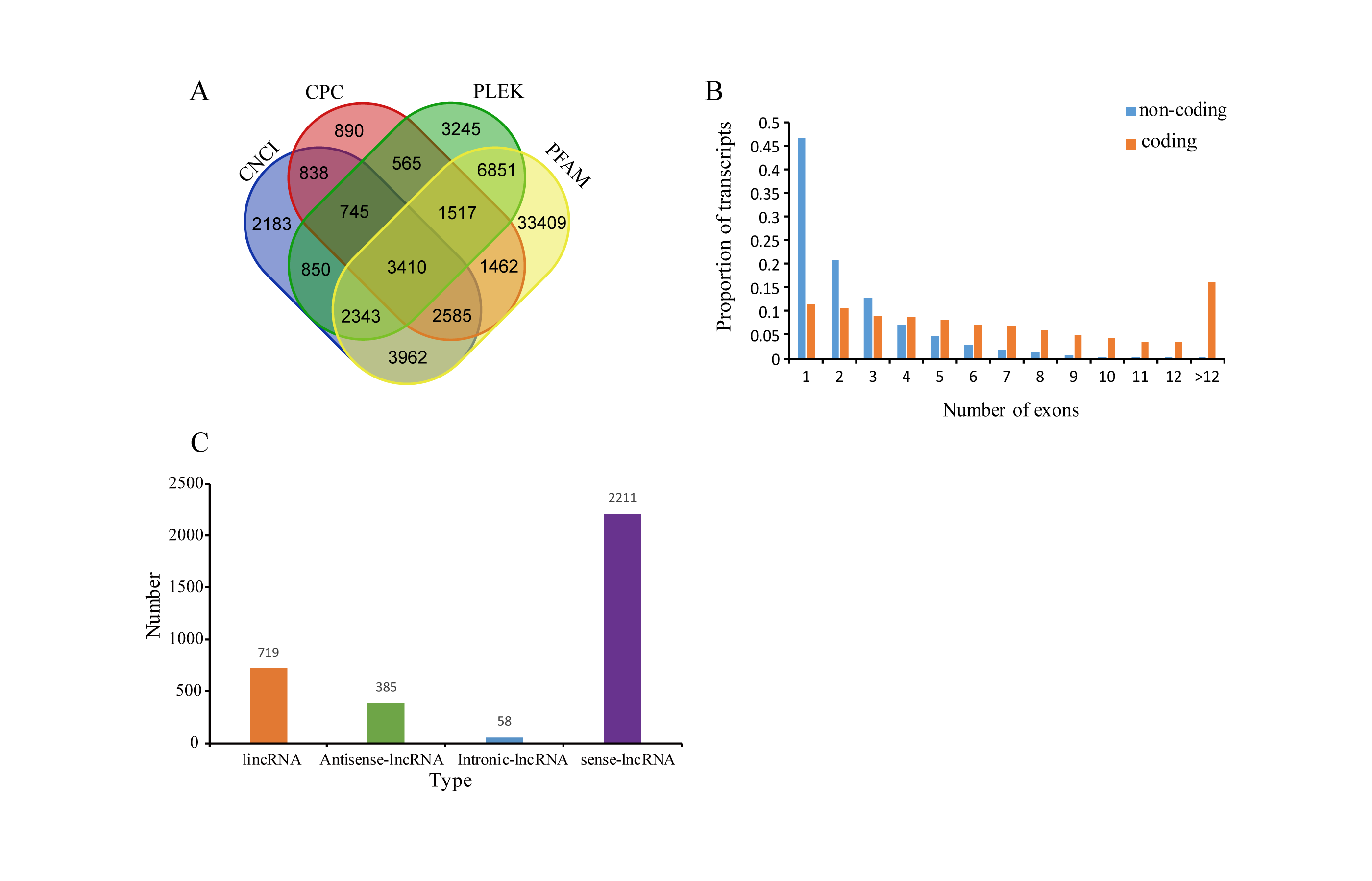

Supplement: Supplementary file 10 [file Image_5.tif]

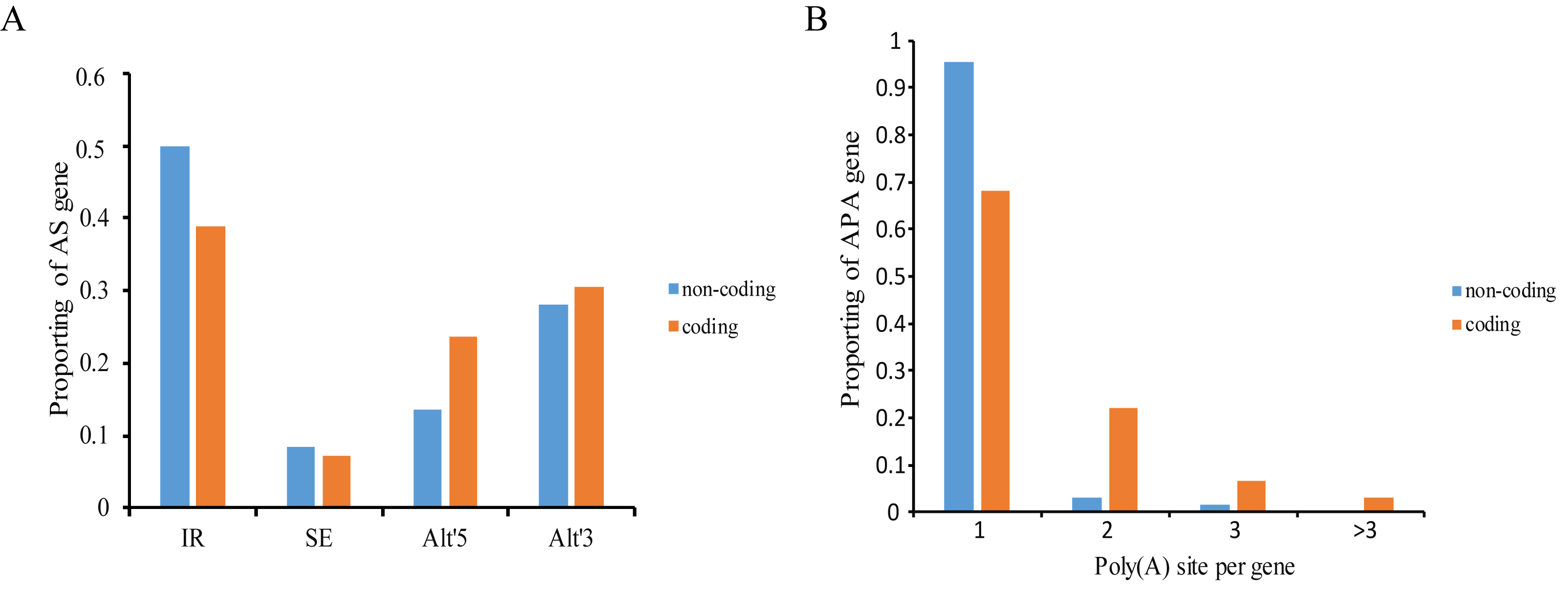

Supplement: Supplementary file 11 [file Image_6.tif]

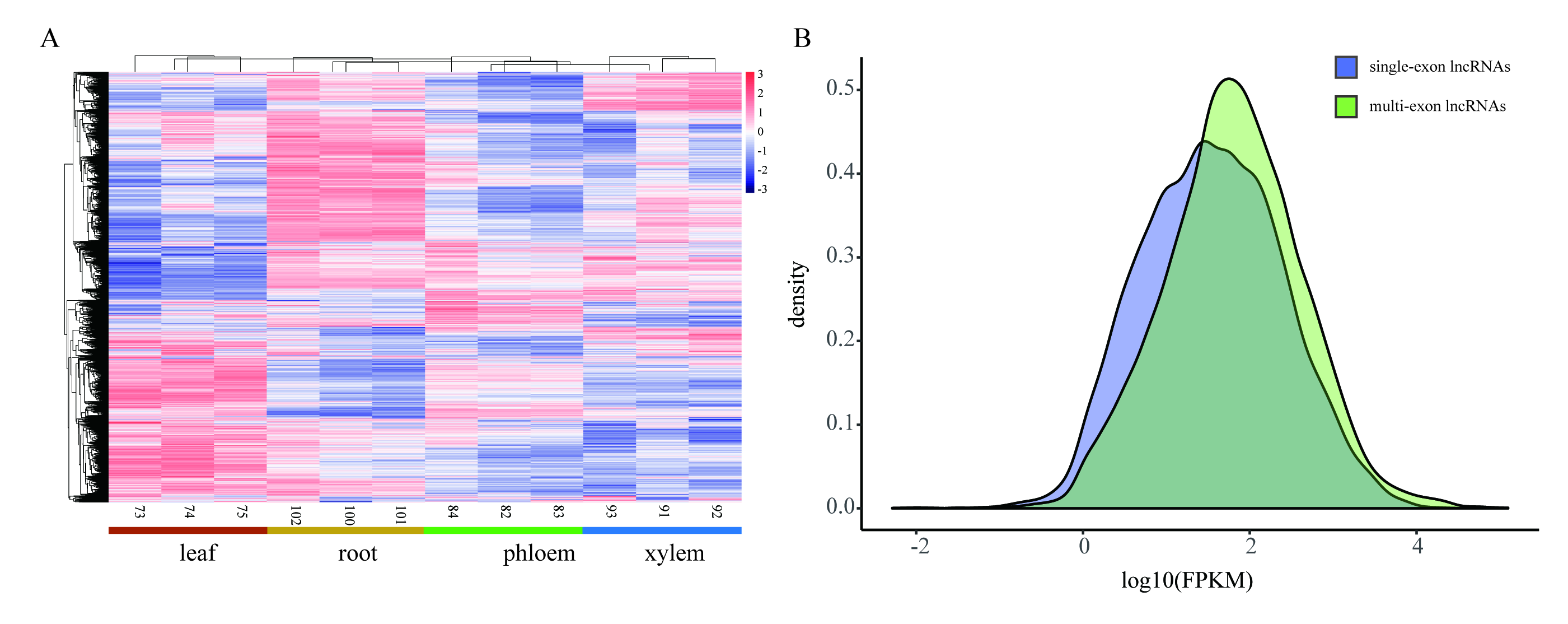

Supplement: Supplementary file 12 [file Image_7.tif]
