# Supplementary material for: Analysis of Alternative Splicing and Alternative Polyadenylation in Populus alba var. pyramidalis by Single-Molecular Long-Read Sequencing
Source: Front Genet. 2020 Feb 7;11:48. doi: 10.3389/fgene.2020.00048 (PMC7020888; doi:10.3389/fgene.2020.00048)
Supplement: Supplementary file 14 [file Table_1.docx]

**Table S1.** Sequencing statistics from the PacBio sequencing

| Sample  ID | cDNA  size | SMRT  Cells | Polymerase  Reads | Post-Filter  Polymerase  Reads | Post-Filter Total  Number of  Subread Bases | Post-Filter  Number of Subread | Post-Filter  Subreads N50 | Post-Filter  Mean Subread  length |
| --- | --- | --- | --- | --- | --- | --- | --- | --- |
| F01 | 1-2kb | 2 | 300,584 | 188,736 | 4,335,251,583 | 2,830,268 | 1,490 | 1,531 |
| F01 | 2-3kb | 3 | 450,876 | 210,474 | 3,766,538,704 | 1,576,152 | 2,629 | 2,389 |
| F01 | 3-6kb | 1 | 150,292 | 103,416 | 2,358,410,813 | 595,711 | 3,885 | 3,958 |
