# Supplementary material for: Analysis of Alternative Splicing and Alternative Polyadenylation in Populus alba var. pyramidalis by Single-Molecular Long-Read Sequencing
Source: Front Genet. 2020 Feb 7;11:48. doi: 10.3389/fgene.2020.00048 (PMC7020888; doi:10.3389/fgene.2020.00048)
Supplement: Supplementary file 15 [file Table_2.docx]

Table S2. The frequency distribution of transcripts length

| FL transcripts length interval | 200-500bp | 500-1000bp | 1000-2000bp | >2000bp | Total |
| --- | --- | --- | --- | --- | --- |
| Number of FL transcripts | 409 | 11255 | 31870 | 61221 | 104755 |
