# Supplementary material for: Analysis of Alternative Splicing and Alternative Polyadenylation in Populus alba var. pyramidalis by Single-Molecular Long-Read Sequencing
Source: Front Genet. 2020 Feb 7;11:48. doi: 10.3389/fgene.2020.00048 (PMC7020888; doi:10.3389/fgene.2020.00048)
Supplement: Supplementary file 16 [file Table_3.docx]

| Type | Transcript number | Mapped gene number | AS gene ratio (%) | APA gene ratio (%) |
| --- | --- | --- | --- | --- |
| Non-coding | 3410 | 970 | 6.08 | 6.70 |
| coding | 101345 | 14996 | 49.49 | 45.55 |

**Table S3.** Comparison of non-coding and coding genes in AS and APA
